# Bisurphate of Lime as a Deodorizer

**Published:** 1871-12

**Authors:** 


Bisulphate of Lime as a Deodorizer.—Dr. J. D. Trask, Astoria, L. I. (*N. Y. Medical Journal*), in a paper on "Carbolic Acid as an Anti-zymotic in Puerperal Diseases," calls attention to the bisulphate of lime as a deodorizer. The excess of sulphurous acid, passing into the atmosphere, comes directly in contact with the offensively odorous particles that have escaped, and at once destroys them. In giving vent to collections of decomposing purulent matter, as in the peritoneal or pleural cavity or in chronic abscesses, cloths wet with this preparation held near the orifice almost entirely prevent the escape into the room of the offensive smell, to the great relief of patient and bystanders. Though the strong solution will destroy fabrics, when properly diluted, as when used for vaginal injections, it neither destroys nor discolors bed-linen.

